# Analysis of mutant allele fractions in driver genes in colorectal cancer – biological and clinical insights

**DOI:** 10.1002/1878-0261.12099

**Published:** 2017-07-20

**Authors:** Rodrigo Dienstmann, Elena Elez, Guillem Argiles, Ignacio Matos, Enrique Sanz‐Garcia, Carolina Ortiz, Teresa Macarulla, Jaume Capdevila, Maria Alsina, Tamara Sauri, Helena Verdaguer, Marta Vilaro, Fiorella Ruiz‐Pace, Cristina Viaplana, Ariadna Garcia, Stefania Landolfi, Hector G. Palmer, Paolo Nuciforo, Jordi Rodon, Ana Vivancos, Josep Tabernero

**Affiliations:** ^1^ Oncology Data Science (ODysSey) Group Vall d'Hebron Institute of Oncology (VHIO) Barcelona Spain; ^2^ Medical Oncology Department Vall d'Hebron University Hospital Vall d'Hebron Institute of Oncology (VHIO) Barcelona Spain; ^3^ Pathology Department Vall d'Hebron University Hospital Universitat Autònoma de Barcelona Spain; ^4^ Stem Cells and Cancer Laboratory Vall d'Hebron Institute of Oncology (VHIO) Barcelona Spain; ^5^ Molecular Oncology Laboratory Vall d'Hebron Institute of Oncology (VHIO) Barcelona Spain; ^6^ Molecular Therapeutics Research Unit Vall d'Hebron Institute of Oncology (VHIO) Barcelona Spain; ^7^ Cancer Genomics Laboratory Vall d'Hebron Institute of Oncology (VHIO) Barcelona Spain

**Keywords:** clonality, colorectal cancer, driver gene, mutant allele fraction

## Abstract

Sequencing of tumors is now routine and guides personalized cancer therapy. Mutant allele fractions (MAFs, or the ‘mutation dose’) of a driver gene may reveal the genomic structure of tumors and influence response to targeted therapies. We performed a comprehensive analysis of MAFs of driver alterations in unpaired primary and metastatic colorectal cancer (CRC) at our institution from 2010 to 2015 and studied their potential clinical relevance. Of 763 CRC samples, 622 had detailed annotation on overall survival in the metastatic setting (OSmet) and 89 received targeted agents matched to *KRAS* (MEK inhibitors), *BRAF* (BRAF inhibitors), or *PIK3CA* mutations (PI3K pathway inhibitors). MAFs of each variant were normalized for tumor purity in the sample (adjMAFs). We found lower adjMAFs for *BRAF*^V^ ^600E^ and *PIK3CA* than for *KRAS*,*NRAS,* and *BRAF* non‐V600 variants. *TP53* and *BRAF*^V^ ^600E^ adjMAFs were higher in metastases as compared to primary tumors, and high *KRAS* adjMAFs were found in CRC metastases of patients with *KRAS* wild‐type primary tumors previously exposed to EGFR antibodies. Patients with RAS‐ or *BRAF*^V^ ^600E^‐mutated tumors, irrespective of adjMAFs, had worse OSmet. There was no significant association between adjMAFs and time to progression on targeted therapies matched to *KRAS*,*BRAF,* or *PIK3CA* mutations, potentially related to the limited antitumor activity of the employed drugs (overall response rate of 4.5%). In conclusion, the lower *BRAF*^V^ ^600E^ and *PIK3CA* adjMAFs in subsets of primary CRC tumors indicate subclonality of these driver genes. Differences in adjMAFs between metastases and primary tumors suggest that approved therapies may result in selection of *BRAF*^V^ ^600E^‐ and *KRAS*‐resistant clones and an increase in genomic heterogeneity with acquired *TP53* alterations. Despite significant differences in prognosis according to mutations in driver oncogenes, adjMAFs levels did not impact on survival and did not help predict benefit with matched targeted agents in the metastatic setting.

**Abbreviations**
CRC: colorectal cancer
MAFs: mutant allele fractions

## 1 Introduction

Tumor next‐generation sequencing (NGS) is routine part of prescreening programs to guide precision cancer therapy. NGS allows the identification of mutations in driver genes in a very sensitive and quantitative manner. The mutant allele fractions (MAFs), also called ‘mutation dose’, represent the number of mutant reads divided by the total number of reads – coverage – at a specific genomic position. In some scenarios, the MAFs of driver genes may have important clinical implications. Examples include the upfront resistance to anti‐EGFR therapies in metastatic colorectal cancer (CRC) with *KRAS* MAFs as low as 1% (Azuara *et al*., 2016; Laurent‐Puig *et al*., 2015), or the positive association between higher *EGFR* ^L858R^ MAFs in lung cancer specimens and longer duration of treatment benefit with gefitinib and erlotinib in the metastatic setting (Ono *et al*., 2014; Zhou *et al*., 2011). More recently, investigators have been tracking MAFs of driver genes to infer mutational timeline and depict dynamic clonal evolution in individual tumors exposed to targeted agents (McGranahan *et al*., 2015; Murtaza *et al*., 2015; Russo *et al*., 2016).

In tissue samples, the MAFs are largely influenced by tumor purity (fraction of neoplastic cells in the sample) and ploidy (either copy number gains or losses of wild‐type/mutant alleles). It is possible to partially adjust the MAF of a mutation by normalizing it to the neoplastic cell content of the sample, which can be named an ‘adjusted MAF’ (adjMAF). Interestingly, when examining NGS results, MAFs do not clearly correlate with tumor purity, and in samples with more than one mutation, adjMAFs are often different among the affected genes (Normanno *et al*., 2015). These findings suggest either coexisting gene mutations and copy number alterations or intratumor genomic heterogeneity, with clonal (truncal) and subclonal driver gene mutations in the same tumor sample.

In many cancer types, including CRC, a comprehensive analysis of driver genes adjMAFs remains to be performed, with particular attention to differences between primary and metastatic lesions, or after exposure to standard therapies. In addition, the potential prognostic effect of a driver gene ‘mutation dose’ in solid tumors has not been investigated in detail. In this manuscript, we present an in‐depth analysis of adjMAFs of driver genes in CRC and estimate their relative clonal and subclonal distribution. We also investigate their potential clinical impact in a large patient cohort with outcome annotation, focusing on survival in the metastatic setting and on the magnitude of response to matched targeted therapies, namely anti‐MEK, anti‐BRAF, and anti‐PI3K agents according to *KRAS*,* BRAF* ^V600E^, and *PIK3CA* adjMAFs, respectively.

## 2 Materials and methods

### 2.1 Mutation analysis and populations of interest

From 2010 to 2015, 763 consecutive patients with metastatic CRC were eligible for targeted sequencing at our institution (Vall d'Hebron Institute of Oncology, VHIO) as part of a molecular prescreening program (MPP) for early drug development. From January 2010 to May 2014, mutation detection and quantification was performed using a multiplex mass spectrometry‐based technology (massARRAY Sequenom^®^ platform, with a 24 oncogene panel of hotspot mutations, including most frequent variants in *KRAS*,* NRAS*,* BRAF,* and *PIK3CA*). Thereafter, we moved to an amplicon‐based NGS technology (MiSeq Illumina^®^ platform, with a 61 oncogene plus tumor suppressor panel, covering most frequently mutated exons of *KRAS*,* NRAS*,* BRAF*,* PIK3CA*,* APC,* and *TP53*). Technical details of mutation analysis are described in Doc S1. Both tests were performed in‐house in our Cancer Genomics Lab under ISO accreditation (UNE‐EN ISO 15189:2013) including mutation detection and quantification. Average sequencing depth was 1000× allowing precise estimates for low MAFs (mutations were called at a minimum MAF of 3%). We used archived formalin‐fixed paraffin‐embedded tissues for sequencing after pathological assessment of neoplastic cell content in the sample by the Molecular Oncology Lab. Tumor purity was defined as the amount of sample occupied by cancer cells and not by surrounding stromal and immune/inflammatory cells, that is, percentage of transformed (neoplastic) cells. In order to mitigate variability, the quantification of neoplastic cells was performed by an experienced pathologist (P.N.) always in the same section used for sequencing, as recently recommended by other groups (Lhermitte *et al*., 2017). A minimum of 20% tumor purity was required for sample processing (resolution at 5% level). Heterogeneous tissue samples were macrodissected for tumor purity assessment and molecular analysis. The calculated adjMAFs (MAF/tumor purity) of driver genes of interest were used to infer clonality of the events. In summary, for oncogenes, the expected adjMAF is close to 0.5 if the event is clonal and < 0.5 if subclonal. For tumor suppressors, as deletion of the wild‐type allele (loss of heterozygosity) is a common genomic event, the expected adjMAFs is > 0.5 if the event is clonal.

Of 763 patients with targeted mutation profiling (molecular population), 622 received oncologic treatment at VHIO and had complete survival annotation (molecular + clinical population). Data curators from the Oncology Data Science (ODysSey) group prospectively extracted this information from medical records in structured clinical–molecular databases. The remaining patients were external referrals to MPP and did not have clinical interventions at our institution. From 622 molecularly and clinically eligible patients, 34 with *KRAS*‐mutated tumors were enrolled in clinical trials with MEK inhibitors, 20 with *BRAF* ^V600E^‐mutated tumors received anti‐BRAF therapy, and 35 with *PIK3CA*‐mutated tumors were treated with an anti‐PI3K agent. Treatment on phase 1 studies continued until disease progression or unacceptable toxicity and was carried out according to the specific requirements of each protocol. Tumor responses were classified as complete, partial, stable disease, or progressive disease as per Response Evaluation Criteria in Solid Tumors (RECIST) v 1.1. Time to progression (TTP) was defined as the time interval from the start of a therapy to its discontinuation for disease progression or death, whichever occurred first (patients with permanent treatment discontinuation for toxicity without evidence of progressive disease were censored at the time of last dose). Overall survival in the metastatic setting (OSmet) was defined as time from first diagnosis of metastasis until death or last follow‐up.

### 2.2 Statistical and ethical considerations

Nonparametric tests (Mann–Whitney *U* and Kruskal–Wallis) were used to cross‐compare adjMAFs of different genes and correlate with variables of interest, such as tissue source (CRC primary vs. metastatic site). Survival analyses (TTP and OSmet) were conducted using the Kaplan–Meier method and compared with the log‐rank test. We constructed univariate and multivariable Cox proportional hazard models for OSmet. The association between TTP and adjMAFs was measured using Pearson's correlation. All tests were two‐sided, and a *P* value < 0.05 was considered statistically significant. Statistical analyses were conducted using r version 3.2.3 (*survival* and *phenoTest* packages). All patients that participated in our institutional MPP signed informed consent form giving investigators access to molecular and clinical data for research purposes. All clinical trials were conducted in accordance with the guidelines of the VHIO Institutional Review Board.

## 3 Results

### 3.1 Biological insights from analysis of driver genes adjMAFs in CRC

As shown in Table 1, sequencing was mostly performed on samples derived from CRC primary tissue. Patients whose metastatic sites were used for profiling had prior exposure to systemic therapies at the time of sample acquisition. This population had sequencing performed exclusively in the metastatic site – unpaired samples. Median tumor purity was 50% (IQR 35–70%), with no significant differences when comparing samples that harbored mutations in driver genes and those wild‐type for the respective genes (*P* > 0.05) or according to the tissue source used for profiling (*P* = 0.16).

**Table 1:** Population characteristics

Molecular population (*n* = 763)		
Mutation analysis	Sequenom^®^	460 (60.3%)
	MiSeq^®^	303 (39.7%)
Tissue source	CRC primary	586 (79.7%)
	Metastasis	149 (20.3%)
	Missing	28
Driver mutation	*KRAS*	365 (47.8%)
	*NRAS*	29 (3.8%)
	*BRAF*	65 (8.5%)
	*PIK3CA*	128 (16.7%)
	*APC* (MiSeq^®^ only)	154 (50.8%)
	*TP53* (MiSeq^®^ only)	191 (63.0%)
Molecular–clinical population (*n* = 622)		
Age at diagnosis	Median (range)	58 years (22–85)
Gender	Male	386 (62%)
	Female	236 (38%)
Stage at diagnosis	Early	252 (41%)
	Metastatic	370 (59%)
CRC primary site	Right	178 (30%)
	Left	260 (45%)
	Rectum	145 (25%)
Number of metastatic sites at diagnosis of metastasis	One	419 (67%)
	Two	163 (26%)
	Three or more	40 (7%)
Metastatic sites	Liver	407 (65%)
	Lung	186 (30%)
	Node	131 (21%)
	Peritoneal	94 (15%)
	Other	57 (9%)
Surgical treatment for metastasis	Any	285 (46%)
	Liver	195 (31%)
	Lung	52 (8%)
	Other sites	57 (9%)
Pharmacological treatment	Oxaliplatin based	599 (97%)
	Irinotecan based	549 (89%)
	Antiangiogenic therapy	342 (55%)
	Anti‐EGFR therapy	285 (46%)
	Anti‐MEK therapy^a^	52 (8%)
	Anti‐BRAF therapy^b^	20 (3%)
	Anti‐PI3K therapy^c^	70 (11%)
	Any other experimental therapy	169 (28%)

^a^RAS mutated or wild‐type, single agent or combo; ^b^ *BRAF* mutated, single agent, or combo; ^c^ *PIK3CA* mutated or wild‐type, single agent, or combo.

**Figure 1:**
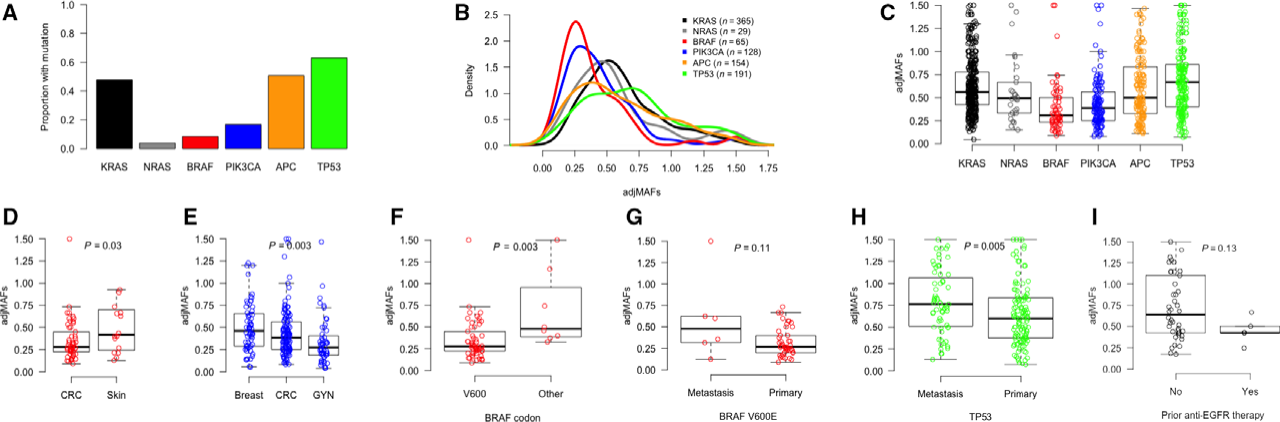
Biological insights into mutant allele fractions adjusted for tumor purity (adjMAFs) in CRC. Proportion of CRC samples with mutations in driver genes (A) and distribution of adjMAFs for the respective genes (B, C). *BRAF*^V^ ^600E^ adjMAFs are higher in melanomas as compared to CRC (D), and *PIK3CA* adjMAFs are higher in breast cancer and lower in gynecological malignancies as compared to CRC (E). *BRAF* adjMAFs are different according to codon affected (higher in non‐V600 mutations as compared to V600; F) and tissue source (trend for higher counts in metastases as compared to CRC primaries; G). *TP53* adjMAFs are also higher in metastases as compared to CRC primaries (H). *KRAS* adjMAFs in metastases of patients with prior exposure to EGFR antibodies (originally *KRAS* wild‐type in the primary tissue) are not significantly different from those without prior targeted treatment (with constitutive *KRAS* mutations in primary tissue) (I).

The prevalence of oncogene mutations in our cohort is depicted in Fig. 1A. The lower prevalence of *APC* mutations (51%) as compared to published literature (around 70%; The Cancer Genome Atlas, 2012) is related to a limited *APC* exon coverage of our NGS panel. Inspection of adjMAFs distribution (Fig. 1B,C) revealed major differences across driver genes. Relative to a simulated normal distribution of adjMAF according to the ‘one‐hit hypothesis’ for oncogenes (median 0.5, IQR 0.25–0.75), we observed significantly lower adjMAFs for *BRAF* (median 0.31, IQR 0.23–0.50; *P* < 0.001) and *PIK3CA* (median 0.38, IQR 0.25–0.56; *P* < 0.001), suggesting a potential subclonality of these genomic alterations. *TP53* adjMAFs (median 0.66, IQR 0.40–0.85) were significantly higher than simulated cohort (*P* < 0.001), indicating a clonal event plus deletion of wild‐type allele in most samples. There were no significant differences between adjMAFs of oncogenes *KRAS* (median 0.56, IQR 0.42–0.77; *P* = 0.66) and *NRAS* (median 0.49, IQR 0.33–0.66; *P* = 0.44) as compared with normal distribution, reinforcing clonality of these events. The same was true for *APC* adjMAFs (median 0.50, IQR 0.32–0.83; *P* = 0.87). Of note, we compared the distribution of oncogene adjMAF according to platform (Sequenom^®^ or MiSeq Illumina^®^) and found no statistically significant differences (*P* > 0.05 for *KRAS*,* NRAS*,* BRAF,* and *PIK3CA*).

Next, we investigated in more detail the potential subclonality of *BRAF* and *PIK3CA* mutations in CRC. Taking advantage of our institutional molecular database, we compared adjMAFs in CRC with those of other malignancies having frequent mutations in these genes. We selected samples from other tumors profiled during the same time period and using similar platforms. As shown in Fig. 1D, *BRAF* ^V600E^ adjMAFs were significantly higher in skin melanomas (median 0.42, IQR 0.25–0.68; *n* = 25; *P* = 0.03) than in CRC. With regard to *PIK3CA* mutations, depicted in Fig. 1E, an intermediate adjMAFs level was seen in CRC as compared to breast cancer (median 0.46, IQR 0.29–0.65; *n* = 64; *P* = 0.002) and gynecological malignancies (median 0.27, IQR 0.19–0.40; *n* = 56; *P* = 0.05). This suggests that *PIK3CA* mutations may be either clonal or subclonal events in CRC. Indeed, when comparing adjMAFs of *PIK3CA* and *KRAS* in CRC samples with co‐occurring mutations (Fig. S1A), we confirmed that potential subclonality of *PIK3CA* mutations is restricted to a subset of CRC tumors (32 of 86 samples [37.2%] with *KRAS*/*PIK3CA* adjMAFs ratio > 1.5).

We then explored differences in adjMAFs of driver oncogenes in CRC according to codon or domain affected and tissue source. As illustrated in Fig. S1B, only *BRAF* adjMAFs vary according to codon affected, being significantly higher in non‐V600 mutations (median 0.48, IQR 0.39–0.85; *n* = 9) as compared to V600 variants (median 0.28, IQR 0.22–0.44; *n* = 56; *P* = 0.003; Fig. 1F). A stratified analysis based on tissue source (Fig. S1C) showed *TP53* adjMAFs significantly higher in metastases (median 0.76, IQR 0.51–1.00; *n* = 63) than CRC primaries (median 0.60, IQR 0.38–0.83; *n* = 126; *P* = 0.005; Fig. 1G), suggesting that *TP53* copy number alterations are more frequent in CRC metastases. A trend for higher *BRAF* ^V600E^ adjMAFs in metastatic sites (median 0.48, IQR 0.32–0.62; *n* = 6) than CRC primaries (median 0.27, IQR 0.21–0.40; *n* = 48; *P* = 0.11; Fig. 1H) was also identified. Four of six *BRAF* ^V600E^ metastatic lesions had prior exposure to anti‐EGFR therapy. Finally, we identified a population with *KRAS*‐mutated metastatic tumors that received treatment with EGFR antibodies prior to biopsy of metastatic site, based on a diagnosis of *KRAS* wild‐type in CRC primary performed outside our institution. As shown in Fig. 1I, *KRAS* adjMAFs of these samples (median 0.43, IQR 0.43–0.50; *n* = 5) were not significantly different from those without prior anti‐EGFR therapy exposure (median 0.6, IQR 0.43–1.1; *n* = 41; *P* = 0.13).

### 3.2 Clinical impact of driver genes adjMAFs in CRC

**Figure 2:**
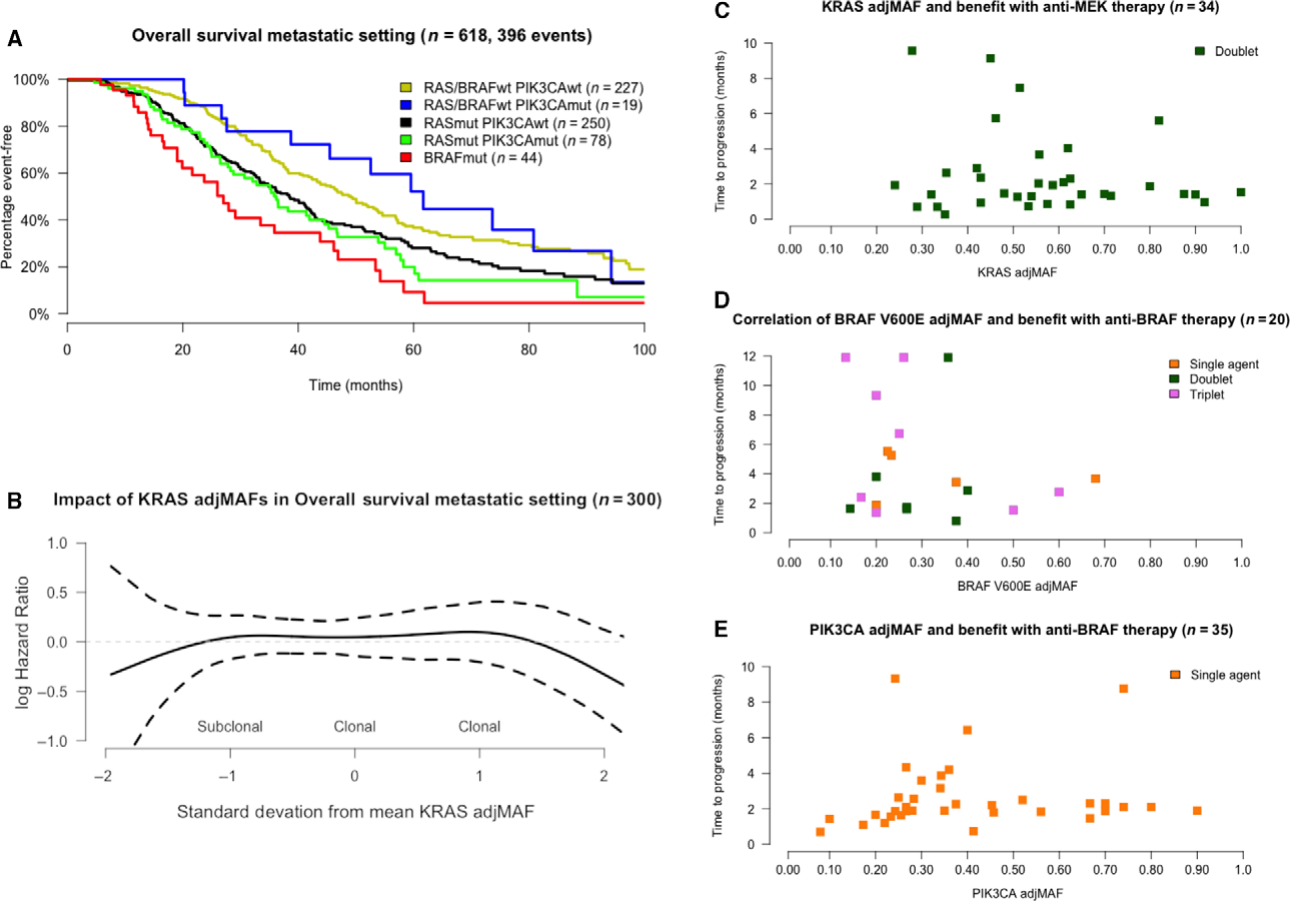
Clinical insights into mutant allele fractions adjusted for tumor purity (adjMAFs) in CRC. Overall survival in the metastatic setting is affected by mutation subgroup (A). The clonality of *KRAS* mutations (as continuous adjMAFs values) does not impact on survival models (B). There is no correlation between *KRAS*,*BRAF*^V^ ^600E^, and *PIK3CA* adjMAFs and TTP on matched targeted therapies, as shown in C–E.

First, we investigated whether the presence of driver oncogene mutations had an impact on prognosis of metastatic CRC patients. As shown in Table 1, this represents an unselected population treated at our institution in the last 6 years, including patients eligible to surgical resection of metastasis during the course of their disease (46%). Most patients had liver metastasis only at diagnosis and were exposed to oxaliplatin‐ and irinotecan‐based chemotherapy plus antiangiogenic agents or anti‐EGFR therapy (if RAS wild‐type). In addition, close to 40% of our patients received experimental agents in early clinical trials in the third‐ or fourth‐line settings. Median follow‐up of patients alive was 38 months (IQR 22–64 months). When aggregating patients in subgroups based on oncogene mutations, as illustrated in Kaplan–Meier curves of Fig. 2A, we observed major differences in median OSmet. In univariate Cox models, detailed in Table 2, we found that patients whose tumors harbored either RAS or *BRAF* mutations had significantly worse OSmet when compared to quadruple wild‐type (*KRAS*,* NRAS*,* BRAF,* and *PIK3CA*) tumors. *PIK3CA* mutations, when having either a more clonal or subclonal pattern of adjMAFs coexisting with RAS mutations, did not negatively impact on OSmet, as illustrated in Fig. S2A**.** Survival was not significantly different according to *KRAS* or *NRAS* codon affected, and only *BRAF* ^V600E^‐mutated tumors had a statistical association with worse OSmet as compared with tumors harboring *KRAS* G12 events, as shown in Fig. S2B and detailed in Table 2. We also found no effect of *KRAS* mutation clonality on survival when considering *KRAS* adjMAFs as a continuous variable (*P* = 0.37), as illustrated in Fig. 2B. Similarly, in the *BRAF* ^V600E^‐mutated population, *BRAF* adjMAFs did not impact on prognosis (*P* = 0.34; results not shown). Next, we constructed a multivariable Cox model with all clinicopathological and molecular covariates that demonstrated statistically significant (*P* < 0.05) association with OSmet in univariate models. The results of our model that included gender, location of CRC primary, the number of metastatic sites, surgical resection of metastasis, RAS mutations, and *BRAF* ^V600E^ mutations are detailed in Table 2. Surgical treatment of metastasis was the strongest determinant of survival in our population, followed by driver oncogene mutations. We also observed a statistically significant effect of CRC primary location on OSmet, with higher risk of death for patients with right‐sided tumors, irrespective of mutations in RAS and *BRAF* ^V600E^.

**Table 2:** Univariate and multivariable Cox models

Univariate models					
Subgroup	No. at risk	No. events	Median OSmet (months) [CI 95%]	Univariate HR [CI 95%]	*P* value (log‐rank)
RAS/*BRAF*wt *PIK3CA*wt	227	143	48.7 [42.4–55.7]	Control	
RAS/*BRAF*wt *PIK3CA*mut	19	12	59.2 [43.9–NR]	0.84 [0.47–1.52]	0.57
RASmut *PIK3CA*wt	250	157	37.0 [32.8–42.2]	1.43 [1.14–1.79]	0.002
RASmut *PIK3CA*mut	78	53	36.5 [28.8–46.8]	1.60 [1.17–2.20]	0.004
*BRAF*mut	44	31	27.2 [19.9–46.2]	2.31 [1.57–3.43]	< 0.001
RASmut *PIK3CA*wt	250	157	37.0 [32.8–42.2]	Control	
RASmut *PIK3CA*mut clonal	44	32	36.8 [27.8–51.4]	1.20 [0.82–1.76]	0.35
RASmut *PIK3CA*mut subclonal	28	19	32.8 [23.7–NR]	1.15 [0.71–1.85]	0.57
*KRAS*mut codon 12	228	156	39.3 [35.1–42.9]	Control	
*KRAS*mut codon 13	46	29	29.5 [22.6–42.8]	1.45 [0.97–2.18]	0.06
*KRAS*mut codon other	29	15	36.2 [29.7–NR]	0.79 [0.46–1.34]	0.38
*NRAS*mut any codon	25	12	51.4 [35.3–NR]	0.68 [0.38–1.23]	0.21
*BRAF*mut V600E	38	25	23.7 [19.0–52.5]	1.70 [1.11–2.60]	0.01
*BRA* mut other	6	6	41.0 [29.1–NR]	1.31 [0.46–1.34]	0.38

Multivariable model		
Variable; *n* = 582, number of events = 371	Multivariable HR [CI 95%]	*P* value (log‐rank)
Male (vs. female)	1.16 [0.93–1.45]	0.18
Number of metastatic sites (2+ vs. 1)	1.29 [1.04–1.62]	0.02
Rectum (vs. left)	1.13 [0.87–1.46]	0.34
Right colon (vs. left)	1.28 [1.01–1.64]	0.05
Surgical resection of metastasis (vs. no)	0.35 [0.28–0.45]	< 0.001
*BRAF* V600E mutation (vs. wt)	1.56 [0.98–2.49]	0.06
RAS mutation (vs. wt)	1.42 [1.15–1.77]	0.001

As prognosis was not affected by clonality of driver oncogenes, we investigated their potential impact on duration of treatment benefit with matched targeted agents. Detailed description of the population and regimens under investigation can be seen in Table 3. Complete or partial responses were only observed in five patients (20%) with *BRAF* ^V600E^‐mutated tumors treated with combination regimens. Median TTP was 1.8 months (CI 95% 1.4–2.4) with MEK inhibitors (given as doublets with another targeted agent) in patients with *KRAS*‐mutated tumors, 3.15 months (CI 95% 1.9–6.7) with BRAF inhibitors (given as single agents, doublets, or triplets) in patients with *BRAF* ^V600E^‐mutated tumors and 2.10 months (CI 95% 1.9–2.6) with PI3K inhibitors (given as single agents) in patients with *PIK3CA*‐mutated tumors. As illustrated in Fig. 2C–E, we found no association between adjMAFs and TTP on matched therapy: Pearson's correlations of −0.12 (CI 95% −0.44 to 0.22; *P* = 0.48), 0.02 (CI 95% −0.46 to 0.43; *P* = 0.94), and 0.05 (CI 95% −0.29 to 0.37; *P* = 0.79) for MEK, BRAF, and PI3K inhibitors, respectively.

**Table 3:** Matched targeted agent population

	Anti‐MEK (*n* = 34)	Anti‐BRAF (*n* = 20)	Anti‐PI3K (*n* = 35)
Gene	*KRAS* mutated	*BRAF* mutated	*PIK3CA* mutated
Variant	G12 = 24 (71%)	V600E = 20 (100%)	Helical = 28 (80%)
	G13 = 6 (17%)		Kinase = 6 (17%)
	Other = 4 (12%)		Other = 1 (3%)
Coexisting mutation	PIK3CA mutation = 12 (35%)	PIK3CA mutation = 4 (20%)	KRAS mutation = 13 (63%)
adjMAF (median, IQR)	0.55 (0.43–0.64)	0.25 (0.20–0.37)	0.34 (0.25–0.54)
Profiling			
Sequenom^®^	31 (91%)	12 (60%)	27 (77%)
MiSeq^®^	3 (9%)	8 (40%)	8 (23%)
CRC primary	28 (82%)	19 (95%)	27 (77%)
Metastasis	6 (18%)	1 (5%)	8 (23%)
Regimen			
Single‐agent inhibitor	0	5 (25%)	35 (100%)
Doublet inhibitor	34 (100%)	7 (35%)	0
	MEK + PI3K = 22 (65%)	BRAF + MEK = 4 (20%)	
	MEK + IGFR1 = 8 (23%)	BRAF + EGFR = 5 (25%)	
	MEK + HER = 4 (12%)		
Triplet inhibitor	0	8 (40%)	0
		BRAF + EGFR + PI3K = 4 (20%)	
		BRAF + EGFR + WNT = 2 (10%)	
		BRAF + EGFR + CDK = 1 (5%)	
		BRAF + EGFR + MEK = 1 (5%)	
Response			
Complete	0	1 (5%)	0
Partial	0	4 (20%)	0
Stable disease	7 (44%)	9 (45%)	11 (31%)
Progressive	19 (56%)	6 (30%)	22 (63%)
NA	0	0	2 (6%)
Discontinuation			
Ongoing	0	1 (5%)	1 (3%)
Progression	31 (91%)	19 (95%)	30 (87%)
Toxicity	3 (9%)	0	2 (5%)
Other	0	0	2 (5%)

## 4 Discussion

NGS of patient tumors has been rapidly incorporated into both prescreening programs and clinical trials over the last years, with the goal of identifying gene alterations that can guide individualized decisions. MAFs of driver genes reflect the genomic complexity of tumors, which may influence prognosis and response to targeted therapies. However, MAFs are frequently underreported or overlooked, which prompted us to investigate whether they have an impact in CRC evolution in the metastatic setting. Our results indicate clonality of RAS mutations and potential subclonality of *BRAF* ^V600E^ mutations and a subset of *PIK3CA* mutations in primary CRC tumors. Normanno *et al*. (2015) also found that in most CRC, the majority of neoplastic cells carry mutant *KRAS* or *NRAS*, while in *BRAF‐* and *PIK3CA*‐mutant cases, only a fraction of neoplastic cells harbor the mutant allele. Alternatively, repetitive copy number alterations co‐occurring with mutations in driver genes could reduce *BRAF* and *PIK3CA* adjMAFs counts. However, the published literature does not support this hypothesis: *BRAF* and *PIK3CA* mutations rarely co‐occur with copy number gains in wild‐type alleles (The Cancer Genome Atlas, 2012; Zack *et al*., 2013). Arm‐level 7q gains (*BRAF* locus) have been described in up to 40% of CRC samples, mainly chromosomally instable tumors lacking *BRAF* mutations (The Cancer Genome Atlas, 2012). In fact, from 20 *BRAF* ^V600E^‐mutated samples in TCGA cohort, only three cases (15%) had coexisting low‐level *BRAF* copy number gains that could possibly explain a lower adjMAF count (The Cancer Genome Atlas, 2012). Similarly, repetitive chromosome 3q gains (*PIK3CA* locus) have not been reported in non‐Asian CRC samples (He *et al*., 2003). Indeed, from 33 *PIK3CA*‐mutated samples in TCGA cohort, only two cases (6%) had coexisting low‐level *PIK3CA* copy number gains (The Cancer Genome Atlas, 2012). Therefore, we believe that *BRAF* ^V600E^ and *PIK3CA* mutations are real subclonal events in subsets of primary CRC tumors.

Other studies have also described clonal–subclonal frequencies of driver alterations in cancer. In a comprehensive analysis of TCGA data in nine solid tumors, McGranahan *et al*. (2015) found a clear tendency for mutations in driver genes to be clonal compared to mutations in noncancer genes. Interestingly, genes involved in the PI3K‐AKT‐mTOR pathway, such as *PIK3CA*, had a higher proportion of subclonal events compared to genes associated with RAS‐MAPK pathway, including *KRAS*,* NRAS,* and *BRAF*. There were clear differences in clonality of *PIK3CA* and *BRAF* ^V600E^ events according to tumor type, and we also identified a more clonal distribution in breast cancer and melanoma, respectively, as compared with CRC. On the other hand, our results suggest distinctive genomic structures according to *BRAF* codon affected, with non‐V600‐mutated tumors harboring a clear clonal pattern. Importantly, none of the studies reported above, including ours, have analyzed MAFs in light of microsatellite instability (MSI), which is associated with hypermutation rates and low copy number alterations (The Cancer Genome Atlas, 2012).

Previous studies have found that the presence of a low fraction of *KRAS*‐mutated cells within primary tumors may provide a reservoir for acquired resistance to EGFR antibodies (Azuara *et al*., 2016; Laurent‐Puig *et al*., 2015). It is surprising that the measurement of MAFs in primary samples correlates with the effects of therapy in the metastatic setting, which suggests that in addition to concordance of mutation events in primary and metastatic samples, the relapsed lesions most likely retain a similar genomic structure, with the same distribution of MAFs. However, we found high *KRAS* adjMAFs in metastatic sites of patients with a previous diagnosis of *KRAS* wild‐type CRC and exposed to anti‐EGFR therapy, suggesting clonal selection of *KRAS*‐mutant cells. Furthermore, differences in MAFs of primary and unmatched metastatic sites for *BRAF* ^V600E^ and *TP53* mutations potentially reflect clonal selection and/or acquired copy number events after therapy. Indeed, the heterogeneity in copy number alterations between matched primary tumors and different metastatic lesions may explain some of the differences in adjMAFs across sites (Sveen *et al*., 2016).

With regard to the clinical implications of driver gene mutations, we observed that RAS and *BRAF* ^V600E^ mutations, irrespective of adjMAFs, have a negative effect on survival in the metastatic setting. These results indicate that even if subclonal, a driver event is biologically relevant. We acknowledge the fact that MSI, a poor prognostic factor in metastatic CRC, has not been taken into consideration in prognostic models (Kim *et al*., 2016). Additionally, we found that the clonality of *KRAS*,* BRAF* ^V600E^, and *PIK3CA* mutations did not predict benefit with matched targeted agents. The negative findings, different from other reports in *EGFR*‐mutated lung cancer with EGFR tyrosine kinase inhibitors (Ono *et al*., 2014; Zhou *et al*., 2011), may be explained by the limited benefit with the targeted therapies explored in our cohort of chemotherapy‐refractory CRC, particularly with MEK and PI3K inhibitors in the *KRAS‐* and *PIK3CA*‐mutant populations, respectively – the latter harboring coexisting *KRAS* mutations known to confer primary resistance to PI3K inhibitors as single agents (Dienstmann *et al*., 2012). For patients treated with BRAF inhibitors, while the poor response to targeted therapy can be explained by constitutive activation of alternative signaling pathways (Prahallad *et al*., 2012), the lack of correlation between *BRAF* ^V600E^ adjMAFs measured in CRC primary tissues and TTP in the metastatic setting may be related to a potential shift in clonality status of *BRAF* ^V600E^ events from primaries to metastases. The association between MAFs of driver gene events identified through circulating tumor DNA (ctDNA) NGS and clinical outcome under targeted therapy should be investigated.

## 5 Conclusion

To conclude, our results suggest that driver gene mutations can be subclonal and even ‘low MAF’ events in NGS tests should be reported. As of today, the absolute MAFs numbers cannot be used to optimize prediction of prognosis or response to matched targeted therapy in CRC. Major limitations of our study include the relatively small and targeted gene panel investigated, lack of copy number data to more precisely define clonality, and the absence of MSI status annotation. From a research perspective, more work needs to be conducted to increase biology understanding before clinical translation. Finally, we believe that analysis of paired primary and metastatic samples from the same patient (longitudinal sampling), with detailed treatment annotation, is crucial to further assess clonality and subclonality patterns of genomic events in cancer. Our work represents a foundation for future efforts assessing the clinical significance of a tumor's genomic structure to guide precision cancer therapy, opening the door for additional investigations on the dynamics of clonal evolution after chemotherapies and targeted drugs.

## Author contribution

RD, IM, ES‐G, CO, MV, FRP, CV, and AG collected, analyzed, and interpreted the data. SL, HGP, PN, and AV involved in molecular data generation and interpretation. EE, GA, IM, ES‐G, TM, JC, MA, TS, HV, JR, and JT involved in clinical data generation and interpretation.

## Disclaimers

Partial results of this study were previously presented at American Society of Clinical Oncology 2016 Annual Meeting as an oral communication in the Colorectal Cancer Clinical Science Symposium, The Clone Wars.

## Supporting information


**Fig. S1.** A subset of samples with co‐occurring *KRAS* and *PIK3CA* mutations has a ‘subclonal’ pattern of *PIK3CA* adjMAFs (defined as *KRAS*/*PIK3CA* adjMAFs ratio > 1.5; A). Driver genes adjMAFs according to codon or domain affected (B) and tissue source (C).

**Fig. S2.** Overall survival in the metastatic setting in *KRAS* mutated colorectal cancer, stratified by co‐occurring *PIK3CA* mutations, either clonal or subclonal events (A). Overall survival in the metastatic setting in patients with tumor mutations in driver genes of the MAPK pathway, stratified by codon affected (B).

**Doc. S1.** Supplementary methods.
