# Supplementary figures and images for: Analysis of mutant allele fractions in driver genes in colorectal cancer – biological and clinical insights
Source: Mol Oncol. 2017 Jul 20;11(9):1263–72. doi: 10.1002/1878-0261.12099 (PMC5579330; doi:10.1002/1878-0261.12099)

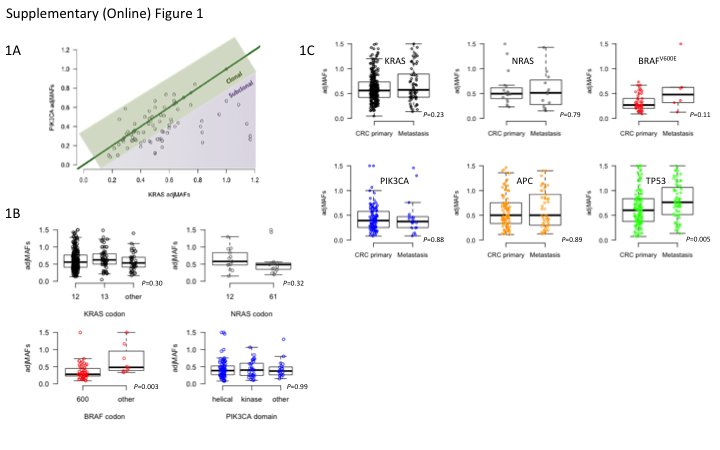

Supplement: Supplementary file 1 — Fig. S1. A subset of samples with co‐occurring KRAS and PIK3CA mutations has a ‘subclonal’ pattern of PIK3CA adjMAFs (defined as KRAS/PIK3CA adjMAFs ratio > 1.5; A). Driver genes adjMAFs according to codon or domain affected (B) and tissue source (C). [file MOL2-11-1263-s001.tiff]

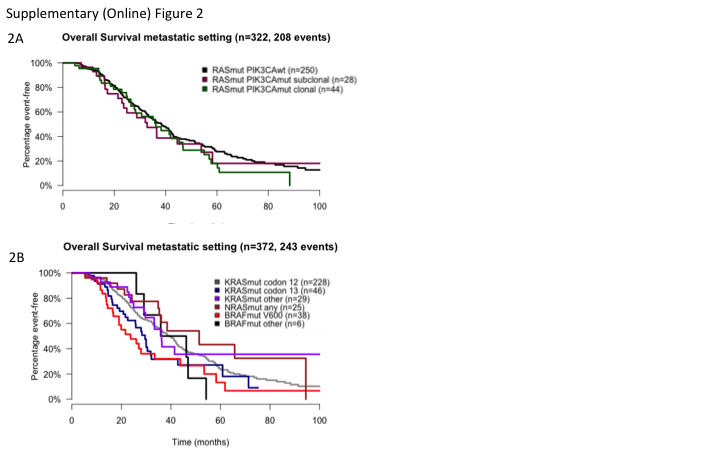

Supplement: Supplementary file 2 — Fig. S2. Overall survival in the metastatic setting in KRAS mutated colorectal cancer, stratified by co‐occurring PIK3CA mutations, either clonal or subclonal events (A). Overall survival in the metastatic setting in patients with tumor mutations in driver genes of the MAPK pathway, stratified by codon affected (B). [file MOL2-11-1263-s002.tiff]
