# Supplementary material for: Analysis of mutant allele fractions in driver genes in colorectal cancer – biological and clinical insights
Source: Mol Oncol. 2017 Jul 20;11(9):1263–72. doi: 10.1002/1878-0261.12099 (PMC5579330; doi:10.1002/1878-0261.12099)
Supplement: Supplementary file 3 — Doc. S1. Supplementary methods. [file MOL2-11-1263-s003.docx]

**Supplementary methods**

DNA extraction was performed from 5X10 Mm sliced sections of FFPE material using the Maxwell FFPE Tissue LEV DNA Purification Kit. Tumor area content was evaluated by a pathologist. A minimum tumor content was set to 20%, in order to ensure subclonal somatic mutations.

**Sequenom**® **- CLIA V2.2 panel**

Somatic mutation profiling of 24 oncogenes was performed by means of the MassARRAY system (Sequenom®) using CLIA v2.2 panel with 273 assays based on IPlex chemistry (Sequenom®).

Briefly, after quantification (nanodrop) and dilution of DNA to a 10 ng/μl concentration, multiplexed PCR was performed in order to amplify the genomic regions that contain the loci to be genotyped (5/Ml volumes containing 0.1 units of Taq polymerase, 20 ng of genomic DNA, 2.5 pmol of each PCR primer and 2.5 pmol of dNTP; thermocycling conditions were as follows: 95 °C for 15 min followed by 45 cycles of 95 °C for 20 s, 56 °C for 30 s and 72 °C for 30 s). Unincorporated dNTPs were deactivated by addition of shrimp alkaline phosphatase (0.3 U) and incubation for 40 min at 37ºC followed by heat inactivation of the enzyme for 5 min at 85ºC. After that, each mutation was analyzed as the single-base extension product of a probe that anneals immediately contiguous to the mutation position (primer extension was carried out adding 5.4 pmol of each primer extension probe, 50 Mmol of ddNTP and 0.5 units of Thermosequenase DNA polymerase to the amplification products; reactions were cycled at 94 °C for 2 min, followed by 40 cycles of 94 °C for 5 s, 50 °C for 5 s and 72 °C for 5 s). After the addition of a cation exchange resin to remove residual salt from the reactions, 7 nl of the purified primer extension reaction were loaded onto a matrix pad (3-hydroxypicoloinic acid) of a Gen II SpectroCHIP (Sequenom®). Gen II SpectroCHIPs were loaded into a matrix-assisted laser desorption/ionization–time of flight (MALDI-TOF) mass spectrometer (Mass ARRAY, Sequenom®) and spectra were obtained for each of the extension products. Data analysis and mutation reports were generated using the Typer Analyzer 4.0 software (Sequenom®). Manual assessing of spectra was performed on each of the reported mutations by the Sequenom® software (mutant allele fractions as low as 5%).

| **CLIA V2.2 PANEL MUTATION LIST** |  |  |
| --- | --- | --- |
| AKT1_E17K | GNAQ_Q209H/L/P/R/Y | PIK3CA_A1046V |
| AKT1_G173R | GNAS_Q227L/R | PIK3CA_C420R |
| AKT1_K179M | GNAS_R201H | PIK3CA_E110K |
| AKT2_E17K | IDH1_R132C/G/H/L/S/V | PIK3CA_E418K |
| AKT2_G175R | IDH2_R172G/K/M/S/W | PIK3CA_E453K |
| AKT3_E17K | KRAS_G12A/V/D/S | PIK3CA_E542K/Q/V/G |
| AKT3_G171R | KRAS_G13C/S/D | PIK3CA_E545A/D/G/K/Q/V |
| BRAF_D594G/V | KRAS_Q61E/H/K/L/P/R | PIK3CA_F909L |
| BRAF_G464E | KRAS_A146T/P/V | PIK3CA_K111N |
| BRAF_G466R/V/E | MET_H1112L/R/Y | PIK3CA_M1043I/V |
| BRAF_G469R | MET_H1124D | PIK3CA_N345K |
| BRAF_K601E/N | MET_M1268T | PIK3CA_P539R |
| BRAF_L597R_1790TG | MET_N375S | PIK3CA_Q060K |
| BRAF_V600A/D/E/G/K/L/M/R | MET_N848S | PIK3CA_Q546E/H/K/L/P/R |
| EGFR_T790M | MET_R988C | PIK3CA_R088Q |
| EGFR_L858R | MET_T1010I | PIK3CA_S405F |
| ERBB2_G309A | MET_Y1248C/H | PIK3CA_T1025A/S |
| ERBB2_H470Q | MET_Y1253D | PIK3CA_Y1021C/H/H |
| ERBB2_I767M | NRAS_G12R/S/C/V/A/D/P/N/Y | PIK3CA_H1047R/L/Y |
| ERBB2_R678Q | NRAS_G13R/S/C/V/A/D/N/Y | PIK3CA_G1049R/S |
| ERBB2_V842I | NRAS_Q61E/H/K/L/P/R | RET_M918T |

**Amplicon-seq VHIO-Card V2 panel**

An initial multiplex-PCR with a proof-reading polymerase was performed on samples using a panel of over 800 primer pairs targeting frequent mutations in oncogenes plus several tumor suppressors, totaling 61 genes.

Indexed libraries were pooled and loaded onto an Illumina MiSeq® instrument and sequencing performed (2X100). Initial alignment was performed with BWA after primer sequence clipping and variant calling performed with the GATK Unified Genotyper and VarScan2 followed by ANNOVAR annotation. SNPs were filtered out with dbSNP and 1000 genome datasets. All detected variants were manually checked. Mutations were called at a minimum mutant allele fraction of 3%.

Genes and regions included in Amplicon-seq VHIO-Card V2 panel:
